# Effect of tDCS Concurrent With VR-Based Robotic Intervention on Hemiplegic Upper Limb Function After Subacute Ischemic Stroke: A Randomized Controlled Study

**DOI:** 10.1155/np/8425060

**Published:** 2025-12-01

**Authors:** Chuan Guo, Ayan Geng, Youxin Sui, Shizhe Zhu, Qinglei Wang, Chaojie Kan, Sheng Xu, Ren Zhuang, Tong Wang, Ying Shen

**Affiliations:** ^1^Department of Rehabilitation Medicine, The First Affiliated Hospital With Nanjing Medical University, Nanjing, China; ^2^School of Rehabilitation Medicine, Nanjing Medical University, Nanjing, China; ^3^Department of Rehabilitation Sciences, The Hong Kong Polytechnic University, Kowloon, Hong Kong; ^4^Department of Rehabilitation Medicine, Changzhou Dean Hospital, Changzhou, China

**Keywords:** functional near-infrared spectroscopy, subacute ischemic stroke, transcranial direct current stimulation, upper limb robot, virtual reality

## Abstract

**Background:**

Upper limb hemiplegia faces the challenge of slow and difficult recovery. A “closed-loop method” based on brain plasticity has been proposed, combining central and peripheral interventions to enhance the upper limb function. Based on the theory, we aimed to investigate the effect of transcranial direct current stimulation (tDCS) concurrent with virtual reality (VR)-based robotic intervention on upper limb recovery and cortical excitability.

**Methods:**

In this single-blinded, randomized, controlled trial, 40 patients with subacute ischemic stroke were recruited and randomized to experimental (tDCS concurrent with VR-based robotic intervention) and control (sham tDCS concurrent with VR-based robotic intervention) groups. All patients received 15 sessions (20 min per day, 5 sessions per week). Outcome measures included the Fugl-Meyer Assessment Upper Limb Scale (FMA-UL), the Action Research Arm Test (ARAT), the Modified Barthel Index (MBI), and functional near-infrared spectroscopy (fNIRS).

**Results:**

All 40 patients completed the intervention, with 34 included in the fNIRS analysis. FMA-UL (*F* = 22.239, *p* < 0.001) and ARAT (*F* = 10.984, *p*=0.002) scores showed significant time-by-group interaction effects. Greater improvements were observed in the experimental group compared to the control group for both FMA-UL (*p* < 0.001) and ARAT (*p*=0.001). MBI scores increased significantly in both groups over time (*F* = 55.415, *p* < 0.001), but the change scores did not differ significantly between groups (*p*=0.369). fNIRS analysis revealed a significant time-by-group interaction effect in the ipsilesional primary motor cortex (M1) (*F* = 4.762, *p*=0.037) and contralesional prefrontal cortex (PFC) (*F* = 10.881, *p*=0.002). Greater increases in activation were found in the experimental group for both ipsilesional M1 (*p*=0.025) and contralesional PFC (*p*=0.002).

**Conclusions:**

Compared with sham tDCS concurrent with VR-based robotic intervention, tDCS concurrent with VR-based robotic intervention can effectively enhance upper limb function and promote activation of ipsilesional M1 and contralesional PFC in subacute ischemic patients with stroke. However, there was no obvious advantage in improving activities of daily life (ADL).

**Trial Registration:** Chinese Clinical Trial Registry: ChiCTR2100047442

## 1. Introduction

Upper limb hemiplegia is a common dysfunction after stroke [1]. Most patients are unable to use the paretic side upper limb to perform simple activities within 6 months after stroke, which may affect living quality and happiness and may cause anxiety and depression [2]. Therefore, improving upper limb function is central to rehabilitation to maximize recovery. However, limited by the fine and complex function of the upper limb and the extremely dense interconnected regulatory network of the brain [3, 4], upper limb hemiplegia still faces the challenge of a slow and difficult recovery.

Recent therapeutic approaches include peripheral interventions such as upper limb robots and virtual reality (VR) technology, and central interventions like transcranial direct current stimulation (tDCS) and repetitive transcranial magnetic stimulation (rTMS) [5, 6]. Nevertheless, the limitations of employing either central or peripheral interventions alone have become increasingly apparent in addressing the growing demands for functional recovery. Based on this, the concept of “closed-loop method” was proposed [7–9]. This theory holds that central intervention can activate the brain and enhance the efficiency of synaptic remodeling, while peripheral intervention strengthens sensory input and feedback to the central nervous system, promoting synapse formation. By combining these interventions, the “closed-loop method” aims to facilitate brain functional remodeling and better promote rehabilitation outcomes [10, 11].

tDCS is a noninvasive brain stimulation technique that modulates cortical excitability and promotes neuroplasticity by delivering low-intensity direct current [12]. Anodal tDCS induces depolarization and enhances cortical excitability, whereas cathodal tDCS causes hyperpolarization and suppresses excitability [13, 14]. This top-down modulation is thought to induce long-term potentiation-like plasticity, potentially mediated by N-methyl-D-aspartate receptors, and may also influence regional cerebral blood flow [12]. Clinical guidelines suggest that anodal tDCS over the ipsilesional M1 is probably effective in improving motor function in patients with subacute stroke (Level B evidence), supporting its use in neurorehabilitation protocols [15]. Due to its portability, low cost, greater tolerability, and compatibility with other interventions, anodal tDCS targeting the ipsilesional M1 is often combined with VR-based robotic training [16, 17]. This creates a closed-loop intervention that engages both central and peripheral systems. In this “closed-loop method,” tDCS exerts top-down neuromodulation to enhance cortical plasticity, thereby creating a favorable neural environment for the sensorimotor feedback induced by VR-based robotic training, which operates through bottom-up mechanisms and contributes to functional reorganization [7–9].

VR-based robotic training integrates hardware and software to achieve an interactive virtual environment, providing patients task-oriented exercises through various games [18]. Multimodal sensory stimulation, including visual, auditory, and tactile stimulation, is provided to enhance patient confidence and encourage high-intensity, repetitive exercise training [19]. Previous studies have shown that VR-based robotic training can significantly enhance functional connectivity between the contralateral M1 and bilateral sensorimotor areas [20]. These findings suggest that VR-based robotic training may facilitate functional recovery through interhemispheric reorganization, consistent with its bottom-up therapeutic mechanism.

To better understand the synergistic effects of tDCS and VR-based robotic training, we designed a randomized controlled trial to evaluate both the clinical efficacy and the underlying mechanisms of cortical activation. Unlike previous studies that focused only on a single intervention or clinical outcomes, this study adopted a synchronous closed-loop design, in which tDCS and VR-based robotic training were administered simultaneously. This approach is more efficient, optimizes central-peripheral interactions, and provides better guidance for clinical application. In addition to clinical outcomes, we used functional near-infrared spectroscopy (fNIRS) to dynamically monitor multi-region cortical activation. fNIRS detects changes in cortical activation by monitoring oxygenation levels in the cortex [21]. Both fNIRS and functional magnetic resonance imaging (fMRI) rely on neurovascular coupling to reflect neuronal activity and brain activation [22]. However, fMRI has superior spatial resolution but is limited by restricted head and body movement, noise, and small testing spaces [23, 24]. In contrast, fNIRS is more portable, less noisy, and more acceptable to patients. Critically, fNIRS allows monitor cortical activation with motor tasks in real time [25, 26].

In this study, we selected the bilateral M1, prefrontal cortex (PFC), and primary sensory cortex (S1) as regions of interest. M1 is the target of anodal tDCS and is pivotal for motor recovery post-stroke [27]. PFC is involved in motor decision-making, preparation, and executive control [28], while the S1 plays a critical role in integrating multisensory feedback during VR-based robotic training [29]. By dynamically monitoring cortical activation in these regions using fNIRS, this study aims to evaluate how the closed-loop intervention promotes neuroplasticity. We hypothesize that tDCS concurrent with VR-based robotic intervention will yield superior clinical effects for hemiplegic upper limb function compared to sham tDCS concurrent with VR-based robotic intervention. Moreover, this intervention is expected to significantly modulate cortical activity in M1, S1, and PFC.

## 2. Methods

### 2.1. Participants

Between June 2021 and June 2022, patients suffering from upper limb hemiplegia following a subacute ischemic stroke were recruited from the Rehabilitation Center in Changzhou Dean Hospital, Changzhou, Jiangsu, China. This study adheres to all CONSORT guidelines and accurately reports the necessary information in accordance with the requirements. A preprint has previously been published [30].

We included patients with a head computed tomography or magnetic resonance imaging examination used to confirm the diagnosis of ischemic stroke, following the Chinese Stroke Association's clinical management guidelines for cerebrovascular disorders [31]: first-ever unilateral ischemic stroke with upper limb hemiplegia, post-stroke duration of 2 weeks to 6 months, and patient age range of 30–80 years. We excluded patients with other neurological or mental illness, severe cognitive impairments that prevented cooperation with the experiment, skull abnormalities, and high muscle tone of the hemiplegic upper limb (Modified Ashworth Scale > 2) [32].

This study was approved by the Changzhou Dean Hospital Human Ethics Committee (CZDALL-2021-001). Each participant provided their written informed consent.

### 2.2. Sample Size

The sample size for this study was calculated using G·Power 3.1 software, employing a randomized controlled experimental design. The primary outcome measure was the Fugl-Meyer Assessment Upper limb scale (FMA-UL). Based on the effect size (Cohen's d value) calculated from previous studies to be 1.207, a two-tailed independent sample *t*-test was chosen [33]. The significance level (α) was 0.05, with a statistical power (1−β) of 0.95, and a 1:1 group allocation ratio. The estimated sample size was 19 cases per group. Considering a 10% attrition rate, the minimum required total sample size was 44 cases.

### 2.3. Randomization and Blinding

A researcher who is not involved in the study will use block randomization to assign 44 patients to the experimental and control groups at a 1:1 ratio. Each block will comprise four patients, totaling 11 blocks. Subsequently, the allocation sequence for each patient will be determined based on the correspondence between computer-generated random numbers and each block. Assignment details will be concealed prior to enrollment, and participants will be unaware of their assigned situation. Given the nature of the intervention, it will not be feasible to blind intervention personnel. However, outcome assessors and data analysts will remain unaware of the allocation.

### 2.4. Study Design and Interventions

**Figure 1:**
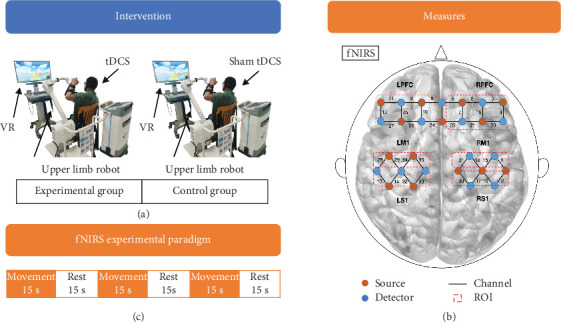
Study design and methods. (a) Study intervention. (b) fNIRS channel setup. The red dots depict detectors, while the blue dots signify light sources. A total of 14 sources and detectors were used, resulting in 35 channels covering seven major regions of interest, namely the left primary motor cortex (LM1), right primary motor cortex (RM1), left prefrontal cortex (LPFC), right prefrontal cortex (RPFC), left primary sensory cortex (LS1), and right primary sensory cortex (RS1). (c) fNIRS experimental paradigm.

The study employed a single-blind, randomized, controlled clinical trial. Patients were randomly allocated to either the experimental or control group using a block randomization method. The demographic information of all patients was collected by the same rehabilitation therapist, including name, age, gender, time after stroke, lesion location, and paretic side. Both groups received basic drug and conventional rehabilitation therapy, consisting of exercise and occupational therapy. Exercise therapy included active and passive training, with the aim of enhancing muscle strength in the hemiplegic upper limb, alleviating muscle tension, and improving joint motion. Reflex inhibition and facilitation techniques were used to correct abnormal movements and promote normal motor patterns. Occupational therapy used a range of therapeutic tools, such as scrub boards, rollers, and multifunctional upper limb training systems, to ease tension in the hemiplegic shoulder blade and reinforce flexion and extension movements in the shoulder, elbow, wrist, and fingers of the hemiplegic upper limb. Both exercise therapy and occupational therapy sessions were conducted for 30 min, once a day, 5 days a week, over a 3-week period. On this basis, the experimental group received tDCS concurrent with VR-based robotic intervention [30]. The control group was treated with sham tDCS concurrent with VR-based robotic intervention. The administration of tDCS was synchronized with the VR-based robotic training (Figure 1a). Both treatment programs included 15 sessions (20 min per session, 5 sessions per week, for 3 weeks).

#### 2.4.1. tDCS

For tDCS, we used a VC-8000C type therapy instrument by Nanjing Wogao Medical Technology Co., Ltd. in Jiangsu, China. Before treatment, the electrodes (5 cm × 7 cm) were inserted into the isosmolar saline gelatin sponge, respectively, and fixed with an elastic bandage. Following the international electroencephalogram (EEG) 10/20 system [34], the anodal tDCS was applied to ipsilesional M1 (C3/C4) on the scalp [35, 36], while cathodal tDCS was positioned over the contralesional superior orbital margin [37]. The tDCS intensity of the experimental group was set at 2.0 mA. In the control group, the two electrodes of the tDCS were applied in the same position as the experimental group. The sham tDCS intensity initially ramped up to 2.0 mA over the first 30 s, before gracefully tapering down to 0 mA over 30 s.

#### 2.4.2. VR-Based Robotic Training

The VR-based robotic training used the EM-BURT02-01 Burt upper limb rehabilitation training system (ESTUN Medical Technology Co., Ltd., Nanjing, Jiangsu, China). It includes a three-dimensional robotic arm and a screen to provide VR environments. The device has been designed with multiple movement modes and game types, such as ocean exploration, shooting, calligraphy, boxing, primarily to assist patients with movement control training, effectively making boring and challenging training interesting. For each patient, the appropriate training modes and exercise items were selected by the specific therapist according to the patients' upper limb function [38]. The VR-based robotic training adapts its assistance level according to the patient's Brunnstrom stage. For Stage II upper limb hemiplegia, it operates in passive movement mode, providing full assistance for shoulder and elbow joint exercises. In Stage III, an assistive movement mode with force-feedback technology offers moderate assistance during task completion. For Stages IV–VI, patients engage in active movement mode, independently controlling the robotic arm for paretic limb training. Throughout the training, the system provides multimodal sensory feedback: visual feedback is provided through VR scenes displayed on the screen, auditory feedback is given via the audio system, and tactile feedback is generated through vibrations at the end of the robotic arm. This combination of sensory stimulation is intended to increase patient interaction and improve the overall training experience.

### 2.5. Outcome Measures

Before and 3 weeks after treatment, upper limb function was assessed using the FMA-UL and the Action Research Arm Test (ARAT). Activities of daily life (ADL) were assessed using the Modified Barthel Index (MBI). fNIRS was applied to monitor cortical excitability while patients flexed and extended the elbow. The FMA-UL served as the primary outcome, while the others were considered secondary outcome. All assessments were performed by the same trained rehabilitation therapist.

#### 2.5.1. Primary Outcome

The FMA-UL assesses reflexes, synergies, range of motion, and fine and gross hand movements, with 33 items scored on a 3-point scale (0–2), giving 66 points [39]. This scale is reliable and valid [40].

#### 2.5.2. Secondary Outcomes

The ARAT is divided into grasping, gripping, pinching, and gross movements, with 19 items and 57 points [41], and has shown good validity and sensitivity [42].

The MBI assesses ADL performance across 10 domains including bowel and bladder care, feeding, grooming, bathing, dressing, toileting, walking, transferring, and climbing stairs, with a total score of 100 points [43]. This index is widely used for evaluating ADL improvements and is considered a reliable tool [44].

We used a multi-channel fNIRS system (NirScan-6000C, Danyang Huichuang Medical Equipment Co., Ltd., Zhenjiang, China) with an 11 Hz sampling rate for data acquisition, with three wavelengths of 730, 808, and 850 nm, and a channel number of 35. The bilateral M1, S1, and PFC were targeted with 14 sources and 14 detectors based on the 10/20 system (Figure 1b).

Since the VR-based robotic training used in this study has shown good training for elbow flexion and extension of the upper limb, and referring to a similar research design [45], we chose the motions of elbow flexion and extension with hemiplegic upper limbs as our fNIRS experiment task. We set the block design as 15 s movement plus 15 s rest, repeated three times [45, 46]. Patients were guided by a therapist to make sure that the procedure was understood before the experiment. The patient applied the fNIRS test cap and sat in a chair, keeping quiet, with the head stable and the stimulator on a table in front of them. Then, patients were instructed by audio cues from the stimulator to repetitively try to flex and extend the elbow for 15 s, and then rest for 15 s (Figure 1c).

### 2.6. Statistical Analysis

#### 2.6.1. fNIRS Data Analysis

Raw data were analyzed using Nirspark (NirScan-6000C). First, the raw near-infrared (NIR) intensities were converted to an optical density signal. Second, irrelevant motion artifacts from the experiment were removed. Third, 0.01–0.1 Hz was used to remove baseline noise as well as possible respiratory and heart rate signals [47]. Fourth, the differential path length factor was set to 6. Fifth, the filtered optical density data were converted to oxy-Hb and deoxy-Hb using the modified Beer–Lambert law [48]. Sixth, the block averaging module of the Nirspark software (with −2 to 0 s as the baseline and 0–30 s as the time paradigm for individual blocks) was used to calculate the total average HbO_2_ concentration in different blocks. Finally, the mean oxygen concentrations of the three blocks were superimposed to obtain the mean value of the blocks. Since the patients in this study had different sides of brain damage, the brain images of patients with right hemisphere injury were flipped to the contralateral hemisphere along the central axis.

#### 2.6.2. Statistical Analysis

Data analysis was conducted using SPSS 25.0. Initially, the Shapiro–Wilk test checked for normality. Normally distributed data were shown as mean ± standard deviation ($\overset{-}{\text{x}}$ ± s), and non-normally data as median and interquartile range (*M* [*P*_25_, *P*_75_]).

Considering both time and group factors, a two-way repeated measures ANOVA was used for data analysis. Non-normal data were square root-transformed to meet the normality assumptions for ANOVA, and the Shapiro–Wilk test performed on the transformed data confirmed normality (*p* > 0.05). Mauchly's test checked sphericity, and if violated, the Greenhouse-Geisser correction was applied. Significant interactions were further analyzed using pairwise *t*-tests for inter-group comparisons and paired *t*-tests for intra-group comparisons, with the Bonferroni-corrected threshold (*p* < 0.0125) derived from dividing 0.05 by four pairwise comparisons.

The score changes of each outcome were also calculated by post-intervention minus pre-intervention to assess improvements. Specifically, *t*-tests were used for normal data, while Mann–Whitney *U* tests were applied for non-normal data.

## 3. Results

### 3.1. Baseline Characteristics of Participants

**Figure 2:**
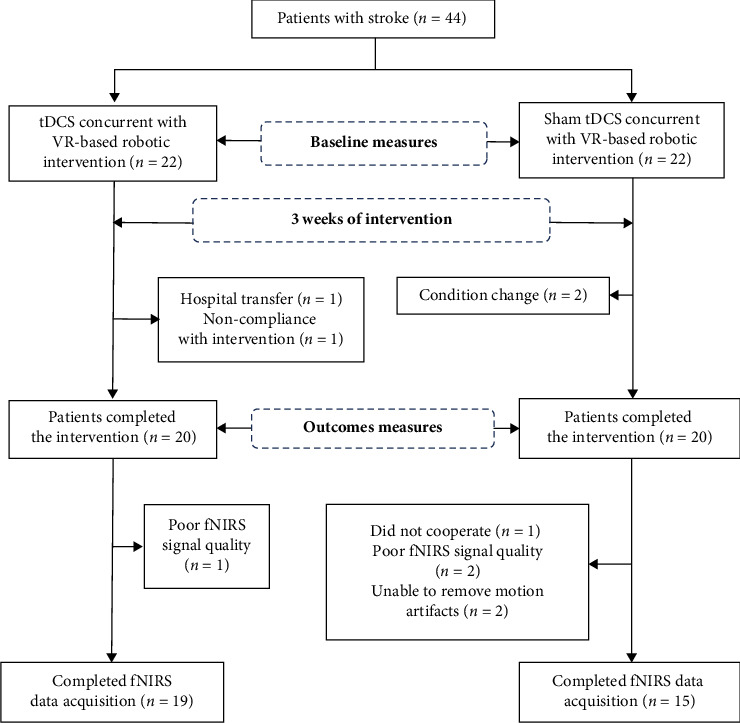
Flowchart of the study.

This study recruited 44 patients. During the experiment, four patients dropped out, including two in the experimental group (one due to hospital transfer, one due to non-compliance with treatment) and two from the control group (due to changes in their condition). Finally, 40 patients completed the experiment (Figure 2).

In the experimental group, one patient could not complete the evaluation due to poor fNIRS signal quality. In the control group, one patient did not cooperate, two patients had poor fNIRS signal quality, and two patients had motion artifacts that could not be removed. In total, 34 patients were included for the fNIRS analysis (Figure 2). Of these, 17 patients with right hemisphere damage (eight from the experimental group, nine from the control group) had their images flipped to left hemisphere damage.

The age, gender, time after stroke, lesion location, and paretic side did not show significant differences between the two groups (all *p* > 0.05) (Table 1).

### 3.2. Effects of the Treatment

#### 3.2.1. FMA-UL

The FMA-UL scores revealed a significant time-by-group interaction effect (*F* = 22.239, *p* < 0.001) and time effect (*F* = 90.238, *p* < 0.001), but no group effect (*F* = 0.880, *p*=0.354). Simple effect analyses indicated no significant differences in scores between groups either pre-intervention (*p*=0.930) or post-intervention (*p*=0.068). However, both groups showed significantly higher scores from pre- to post-intervention (*p* < 0.0125), regarding the change scores of FMA-UL. Further analysis of the change scores demonstrated that the experimental group exhibited significantly greater change scores compared to the control group (*p* < 0.001).

#### 3.2.2. ARAT

The ARAT scores revealed a significant time-by-group interaction effect (*F* = 10.984, *p*=0.002) and time effect (*F* = 49.975, *p* < 0.001), but no group effect (*F* = 0.346, *p*=0.560). Simple effect analyses indicated no significant differences in scores between groups either pre-intervention (*p*=0.779) or post-intervention (*p*=0.355). However, both groups showed significantly higher scores from pre- to post-intervention (both *p* < 0.0125), regarding the change scores of ARAT. Further analysis of the change scores demonstrated that the experimental group exhibited significantly greater change scores compared to the control group (*p*=0.001).

#### 3.2.3. MBI

The MBI scores revealed no significant time-by-group interaction effect (*F* = 0.564, *p*=0.457) or group effect (*F* = 0.509, *p*=0.480). However, we found a statistically significant time effect (*F* = 55.415, *p* < 0.001), and both groups showed significantly higher scores from pre- to post-intervention (*p* < 0.0125). However, the change scores did not differ significantly between groups (*p*=0.369).

**Figure 3:**
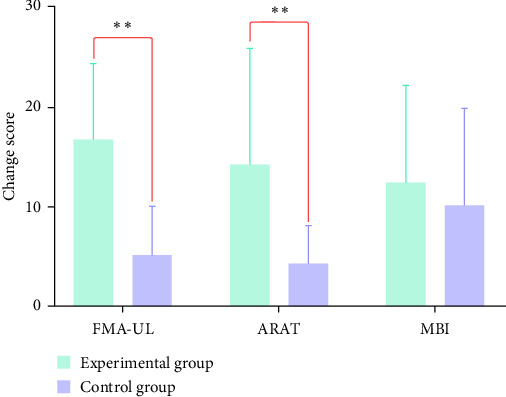
The differences in FMA-UL, ARAT, and MBI scores between the two groups. *⁣*^*∗∗*^*p* < 0.05, significant between-group difference. ARAT, action research arm test; FMA-UL, Fugl-Meyer assessment upper limb scale; MBI, modified Barthel Index.

Tables 2 and 3 present pre- and post-intervention scores and two-way repeated measures ANOVA results for FMA-UL, ARAT, and MBI.  Figure 3 illustrates the differences in FMA-UL, ARAT, and MBI scores between groups.

#### 3.2.4. fNIRS

The analysis of the ipsilesional M1 revealed a significant time-by-group interaction effect (*F* = 4.762, *p*=0.037), with no significant time effect (*F* = 0.101, *p*=0.753) or group effect (*F* = 0.065, *p*=0.801) were not significant. Simple effect analyses indicated no significant differences in activation values between groups pre-intervention (*p*=0.537) or post-intervention (*p*=0.083). Neither group exhibited significant changes in activation values from pre- to post-intervention (both *p*  > 0.0125). Further analysis of change activation values demonstrated that the experimental group had significantly greater increases in activation than the control group (*p*=0.025).

The analysis of the contralesional PFC revealed a significant time-by-group interaction effect (*F* = 10.881, *p*=0.002), with no significant time effect (*F* = 0.662, *p*=0.422) or group effect (*F* = 0.107, *p*=0.745). Simple effect analyses indicated no significant differences in activation values between groups pre-intervention (*p*=0.128) or post-intervention (*p*=0.358). The experimental group showed no significant difference in activation values from pre- to post-intervention (*p* > 0.0125), although a trend toward increased activation was noted, whereas the control group exhibited a significant decrease over the same period (*p* < 0.0125). Further analysis of the change activation values demonstrated that the experimental group had significantly greater increases in activation than the control group (*p*=0.002).

The analysis of the contralesional M1, ipsilesional PFC, ipsilesional S1, and contralesional S1 revealed no significant time-by-group interaction effect, time effect, or group effect.

**Figure 4:**
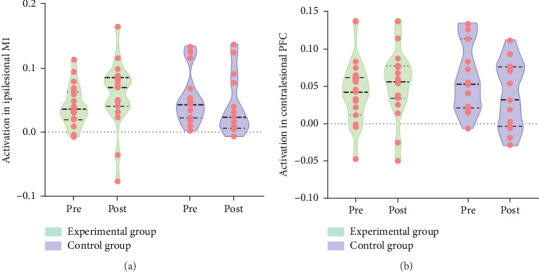
Scatter plots of HbO_2_ in the experimental group and control group. The HbO_2_ concentration changes in the (a) ipsilesional M1 and (b) contralesional PFC of two groups. M1, primary motor cortex; PFC, prefrontal cortex.

Tables 4 and 5 summarize HbO_2_ levels and two-way ANOVA results for M1, PFC, and S1.  Figure 4 illustrates the HbO_2_ changes in the ipsilesional M1 and contralesional PFC of two groups.

## 4. Discussion

We found that, after 3 weeks of tDCS concurrent with VR-based robotic intervention, there was a significantly different pattern in the enhancement of upper limb function between the groups. Simultaneously, fNIRS data demonstrated a significant increase in ipsilesional M1 and contralesional PFC activation in the experimental group relative to the control group. In comparison to sham tDCS concurrent VR-based robotic intervention, this suggests that tDCS concurrent with VR-based robotic intervention can effectively promote the change of cortical excitability and enhance upper limb function in subacute stroke patients.

Stroke destroys the balance of the bilateral hemisphere and activates brain plasticity [33], making the promotion of neural plasticity a key focus in stroke rehabilitation. On the basis of the “closed-loop method” [7, 9], we combined tDCS with VR-based robotic training to observe whether synchronous intervention could better restore upper limb function. After anodal tDCS, cortical facilitation is enhanced and local inhibition is weakened, which may initiate neuroplasticity and motor learning effects, thus promoting the recovery of motor function [14, 49]. Meta-analysis shows that tDCS combined with rehabilitation significantly improves arm and hand function after stroke, outperforming sham tDCS or tDCS alone [50]. In this study, tDCS was combined with VR-based robotic training, which can provide a rich virtual environment and repeated reinforcement of virtual actions that enable multidimensional training of the shoulder and elbow joints through goal orientation in a common environment of daily life [51]. At the same time, integrated visual, auditory, and tactile feedback encourages task completion, enhances activation of the ipsilesional hemisphere, promotes M1 plasticity and reorganization, and facilitates more effective motor learning [52–54].

In short, tDCS concurrent with VR-based robotic intervention aimed to enhance the single intervention effect, maximizing the recovery of hemiplegic upper limb function. Yao et al. randomly assigned 40 subacute stroke patients to two groups: an experimental group received tDCS concurrent with VR intervention, and a control group received sham tDCS concurrent with VR intervention. The difference between the pre-intervention and post-intervention scores demonstrated that the experimental group's FMA-UL, ARAT, and BI scores improved significantly compared to the control group. It is suggested that tDCS concurrent with VR is more effective than VR alone [33]. Our investigation yielded similar results; however, the MBI difference was not statistically significant, which might be related to the use of MBI scores in this study. Its items were more detailed than the BI score, and it was easier to reflect the improvement of ADL [55]. Straudi et al. [56] found that, when compared to before an intervention, the FMA-UL score of tDCS concurrent with upper limb robotic intervention (experimental group) and sham tDCS concurrent with upper limb robotic intervention (control group) improved after the intervention, but the difference between groups was not statistically significant. The main difference between this study and ours is that the disease course and lesion location of the included patients are inconsistent, and there is heterogeneity. Our patients were those with subacute subcortical strokes who may have had greater functional recovery. This also suggests that the course of the disease and the location of the lesion may be important factors affecting the therapeutic effect.

Studies have used anodal tDCS to stimulate the ipsilesional M1 to boost M1 excitability and promote neuroplasticity [50, 57]. Neuroimaging studies using fMRI and EEG indicate that tDCS not only activates M1 but also modulates interconnected regions, such as the premotor and somatosensory cortex [58, 59]. Consistently, we observed increased activation of ipsilesional M1 and contralesional PFC after anodal tDCS concurrent with VR-based robotic intervention. M1 is responsible for controlling and performing motor tasks, and PFC integrates visual information with motor responses [60, 61]. These excitability changes likely have two causes. Firstly, VR-based robotic training requires patients to understand, judge, select, and execute task information, increasing cognitive load and enhancing M1 and PFC activation [62, 63]. Secondly, anodal tDCS may activate task-related brain networks [64]. In the experimental group, anodal tDCS of ipsilesional M1 not only increased ipsilesional M1 activation but also enhanced activation in task-related brain regions like PFC. Moreover, our research findings align with the “closed-loop method” [7–9]. The VR-based robotic training provides multimodal sensory input, creating a sensorimotor feedback loop that anodal tDCS reinforces, promoting sensorimotor integration and brain circuit reorganization [19, 65]. These findings suggest that the increased activation of ipsilesional M1 and contralesional PFC in the experimental group may reflect the synergistic effects of central and peripheral interventions, whereas the limited activation in the control group and its modest impact on neuroplasticity may explain the nonsignificant main effect of time. This also indirectly highlights the pronounced role of neuromodulation in promoting neuroplasticity.

The strength of this study is that it provides objective evidence for tDCS concurrent with VR-based robotic intervention from the aspect of brain imaging and complements the clinical scale, which helps to more accurately reflect the effectiveness of rehabilitation treatment on the restoration of upper limb function in stroke patients. However, the study also has several limitations. First, the size of the sample is small. Second, due to the impact of the epidemic, the decrease in patient admissions has made it difficult to establish a blank control group. Therefore, we are facing challenges in verifying whether the improvement in patient functional status also includes natural recovery from the illness. Third, the effects of different intervention timing and frequency were not explored, which may influence the cumulative impact of training on upper limb function and cortical excitability [66, 67]. Fourth, fNIRS task acquisition task only selected elbow flexion and extension, aiming to match the VR-based robotic training action. This task may not fully capture complex cortical responses related to upper limb movements. Future studies should address these limitations by including larger, more diverse samples and implementing stratified analyses based on stroke severity. In addition, varying the intervention timing and frequency, as well as incorporating valid motor tasks during fNIRS, may help clarify the mechanisms of action and better reflect outcomes.

## 5. Conclusions

Compared with the sham tDCS concurrent with VR-based robotic intervention, there was a significant enhancement in upper limb function after tDCS concurrent with VR-based robotic intervention; however, there was no obvious advantage in improving ADL. Compared with the sham tDCS concurrent with VR-based robotic intervention, the activation of the ipsilesional M1 and the contralesional PFC were significantly changed after the tDCS concurrent with VR-based robotic intervention.

## Figures and Tables

**Table 1:** Demographics and characteristics of the stroke patients in the two groups.

Characteristic	Experiment group (*n* = 20)	Control group (*n* = 20)	*p* Value
Age (years), mean (SD)	61.10 (10.62)	62.35 (12.14)	0.731^a^
Gender (male/female)	17/3	16/4	0.451^b^
Time after stroke (*M* (*P*_25_, *P*_75_))	28.50 (17.25, 49.25)	30.50 (16.25, 55.00)	0.968^c^
Stroke lesion locations Cortical Subcortical Mixed			1.000^b^
	2	1	
	14	15	
	4	4	
Paretic side			0.752^b^
Left	9	11	
Right	11	9	

^a^ *t* value calculated by *t* test.

^b^Fisher's exact test.

^c^ *z* value calculated by Mann–Whitney *U* test.

**Table 2:** Pre- and post-intervention FMA-UL, ARAT, and MBI scores in two groups.

Outcome	Experimental group (*n* = 20)	Control group (*n* = 20)
FMA-UL		
Pre	32.30 ± 15.18	32.75 ± 16.99
Post	48.05 ± 15.16	38.05 ± 18.34
Change	15.00 (9.00, 21.00)	4.50 (1.25, 8.00)
ARAT		
Pre	12.50 (3.25, 30.00)	14.00 (3.25, 43.00)
Post	35.50 (11.50, 50.00)	23.50 (4.00, 49.75)
Change	12.00 (5.00, 23.00)	4.00 (0.50, 6.00)
MBI		
Pre	71.50 ± 14.36	68.55 ± 23.44
Post	84.05 ± 13.02	78.80 ± 22.12
Change	9.50 (6.00, 16.50)	9.00 (3.00, 13.75)

*Note:* Normally distributed data were shown as mean ± standard deviation ($\overset{-}{\text{x}}$ ± s), and non-normally data as median and interquartile range (*M* (*P*_25_, *P*_75_)).

Abbreviations: ARAT, action research arm test; FMA-UL, Fugl-Meyer assessment upper limb scale; MBI, modified Barthel Index.

**Table 3:** Results of two-way repeated measures ANOVA for FMA-UL, ARAT, and MBI.

Two-way repeated measures ANOVA	Time-by-group interaction effect		Time effect		Group effect	
	*F* Value	*p* Value	*F* Value	*p* Value	*F* Value	*p* Value
FMA-UL	22.239	<0.001	90.238	<0.001	0.880	0.354
ARAT	10.984	0.002	49.975	<0.001	0.346	0.560
MBI	0.564	0.457	55.415	<0.001	0.509	0.480

Abbreviations: ARAT, action research arm test; FMA-UL, Fugl-Meyer assessment upper limb scale; MBI, Modified Barthel Index.

**Table 4:** Pre- and post-intervention HbO_2_ levels during the elbow flexion task in two groups.

ROI	Experimental group (*n* = 19)	Control group (*n* = 15)
Ipsilesional M1		
Pre	0.041 ± 0.032	0.052 ± 0.041
Post	0.058 ± 0.053	0.040 ± 0.046
Change	0.017 ± 0.049	−0.012 ± 0.018
Contralesional M1		
Pre	0.043 ± 0.045	0.049 ± 0.046
Post	0.050 ± 0.043	0.048 ± 0.046
Change	0.008 ± 0.056	−0.001 ± 0.038
Ipsilesional PFC		
Pre	−0.036 ± 0.046	0.051 ± 0.058
Post	0.039 ± 0.044	0.037 ± 0.040
Change	0.003 ± 0.030	−0.014 ± 0.045
Contralesional PFC		
Pre	0.039 ± 0.040	0.062 ± 0.046
Post	0.053 ± 0.044	0.039 ± 0.044
Change	0.014 ± 0.037	−0.023 ± 0.026
Ipsilesional S1		
Pre	0.064 ± 0.044	0.051 ± 0.042
Post	0.069 ± 0.064	0.054 ± 0.040
Change	0.005 ± 0.064	0.004 ± 0.026
Contralesional S1		
Pre	0.062 ± 0.047	0.046 ± 0.039
Post	0.046 ± 0.046	0.049 ± 0.042
Change	−0.016 ± 0.046	0.003 ± 0.042

*Note:* Normally distributed data were shown as mean ± standard deviation ($\overset{-}{\text{x}}$ ± s). M1, primary motor cortex; S1, primary sensory cortex.

Abbreviations: PFC, prefrontal cortex; ROI, regions of interest.

**Table 5:** Results of two-way repeated measures ANOVA for M1, PFC, and S1.

Two-way repeated measures ANOVA	Time-by-group interaction effect		Time effect		Group effect	
	*F* Value	*p* Value	*F* Value	*p* Value	*F* Value	*p* Value
Ipsilesional M1	4.762	0.037	0.101	0.753	0.065	0.801
Contralesional M1	0.259	0.614	0.160	0.692	0.036	0.851
Ipsilesional PFC	1.713	0.200	0.753	0.392	0.185	0.670
Contralesional PFC	10.881	0.002	0.662	0.422	0.107	0.745
Ipsilesional S1	0.005	0.944	0.234	0.632	0.978	0.330
Contralesional S1	1.596	0.216	0.704	0.408	0.267	0.609

*Note:* M1, primary motor cortex; S1, primary sensory cortex.

Abbreviation: PFC, prefrontal cortex.

## Data Availability

The data that support the findings of this study are available upon request from the corresponding author. The data are not publicly available due to privacy or ethical restrictions.
